# Understanding the performance and mechanism of Mg-containing oxides as support catalysts in the thermal dry reforming of methane

**DOI:** 10.3762/bjnano.9.108

**Published:** 2018-04-13

**Authors:** Nor Fazila Khairudin, Mohd Farid Fahmi Sukri, Mehrnoush Khavarian, Abdul Rahman Mohamed

**Affiliations:** 1 School of Chemical Engineering, Universiti Sains Malaysia, 14300 Nibong Tebal, Pulau Pinang, Malaysia

**Keywords:** carbon formation, catalyst development, dry reforming of methane, magnesium catalyst, mechanism

## Abstract

Dry reforming of methane (DRM) is one of the more promising methods for syngas (synthetic gas) production and co-utilization of methane and carbon dioxide, which are the main greenhouse gases. Magnesium is commonly applied in a Ni-based catalyst in DRM to improve catalyst performance and inhibit carbon deposition. The aim of this review is to gain better insight into recent developments on the use of Mg as a support or promoter for DRM catalysts. Its high basicity and high thermal stability make Mg suitable for introduction into the highly endothermic reaction of DRM. The introduction of Mg as a support or promoter for Ni-based catalysts allows for good metal dispersion on the catalyst surface, which consequently facilitates high catalytic activity and low catalyst deactivation. The mechanism of DRM and carbon formation and reduction are reviewed. This work further explores how different constraints, such as the synthesis method, metal loading, pretreatment, and operating conditions, influence the dry reforming reactions and product yields. In this review, different strategies for enhancing catalytic activity and the effect of metal dispersion on Mg-containing oxide catalysts are highlighted.

## Review

### Introduction

Global warming is a present critical issue resulting from the excessive production of greenhouse gases, particularly carbon dioxide and methane (CO_2_ and CH_4_). This phenomenon is attributed to human dependence on fossil fuels to meet the high energy demands of the rapidly increasing world population. Therefore, one of the solutions to mitigate the emission of greenhouse gases is to utilize these gases to yield new products, such as hydrogen or syngas (synthetic gas) [1–4]. Three principal technologies used for the production of syngas are steam reforming [5–7], partial oxidation [8–10], and dry reforming of methane (DRM) [11–13]. Among these technologies, DRM has attracted a growing body of research because of its potential to simultaneously utilize both carbon dioxide and methane gases. DRM is the most promising approach because the H_2_/CO ratio for this reaction favors the Fischer–Tropsch synthesis [14–15]. DRM is yet to be commercialized on an industrial scale, as this method still has several issues and limitations, including the following: (i) the endothermic nature of the DRM reaction, (ii) catalyst deactivation, and (iii) the H_2_/CO product ratio is lower than unity due to the occurrence of a reverse water–gas shift (RWGS) reaction, CO_2_ + H_2_ ↔ CO + H_2_O. However, the major obstacle in implementing this technology is catalyst deactivation, which is defined as the susceptibility to carbon deposition and sintering of both support and active metal particles [16–18]. The loss of catalytic activity or selectivity over time of production affects the cost of catalyst replacement, process shutdown, and product quality and quantity. Numerous researchers have reported that noble metal-based catalysts, such as Pt, Rh, Pd, Ru, and Ir, exhibit high activity and resistance toward carbon formation [19–21]. However, these noble metals are associated with high cost and low availability, so non-noble metals, such as Ni [18,22–24], Fe [25–28], and Co [29–30] are most often used. Among the non-noble metals, Ni has been frequently chosen as an active catalyst metal owing to its vast availability, excellent activity, substantial redox characteristic, and relatively low cost [31]. However, Ni-based catalysts suffer from significant carbon deposition, which leads to carbon deactivation [32–33]. Recently, numerous attempts have been made to minimize carbon deposition on the catalyst surface in a DRM reaction. A high dispersion of active metal over the support is an effective approach to decrease carbon deposition by producing catalysts of small particle size [19,28,31,34–40]. In addition, a high dispersion of metal particles enhances the interaction between metal and support, consequently reducing carbon deposition [40–44].

One other alternative for inhibiting carbon deposition, as suggested by researchers, is the application of a second metal oxide, such as alkali or alkaline earth metal oxides, to the DRM catalyst to alter the catalyst acidity [39,45–48]. Numerous publications have reported on the ability of Mg-containing oxide catalysts to decrease the rate of carbon formation in a DRM reaction [18–19,49–54]. MgO exhibits superior surface and catalytic characteristics as a catalyst support combined with a high dispersion of small active particles for suppressing carbon formation [55]. The decreased carbon formation in DRM is due to the high basicity of MgO to capture CO_2_, thereby accelerating the overall reaction [34,49,51]. Furthermore, its characteristics such as high thermal stability, high melting point, and low cost make MgO a promising component in endothermic reactions, especially in the DRM process [18,34,49].

Therefore, this review discusses the research regarding the DRM reaction over Mg-containing oxide catalysts as conducted by numerous researchers to assist in the reaction for decreasing carbon formation and to improve catalyst activity, selectivity, and stability. To date, an incorporated mechanism for carbon deposition and removal has yet to be proposed and is still being debated among researchers. The present review mainly focuses on the mechanism of DRM, carbon formation and removal, the role of Mg-containing oxide catalysts in resistance to catalyst deactivation, Mg-containing oxide catalyst development, and the effect of reaction conditions on catalyst performance.

### Thermodynamic analysis

Thermodynamic analysis of a reaction is important for the theoretical study on the behavior of the process and the possible reaction conditions. A thermodynamic study could provide a basis for experimental investigation of the DRM reaction because the chemical reactions are conducted under equilibrium conditions [13,56]. Pressure, temperature and the composition of reactants (CH_4_ and CO_2_) are emphasized the most in thermodynamic studies using Gibbs free energy minimization technique [13,57–58].

#### Effect of temperature and pressure in DRM

In a DRM reaction, the conversion of CH_4_ and CO_2_ increases with an increase in temperature. This is because the DRM reaction is endothermic. In addition the reaction temperature in the DRM is the most important factor compared to pressure and reactant composition. As the temperature increases, the reaction approximately reaches complete conversion and equilibrium is achieved. Nikoo et al. [56] investigated the effect of temperature on the equilibrium conversion of CH_4_ and CO_2_ in a DRM reaction using Gibbs free energy minimization method. They showed that at low temperatures, *T*_rxn_ < 800 °C (1073 K), the equilibrium conversion of CH_4_ was different for different ratios of CO_2_/CH_4_. However, when the temperature increased, *T*_rxn_ ≥ 800 °C, for ratios of 2 and 3, the equilibrium conversion of CH_4_ was 100% and for ratios 0.5 and 1, the conversion reached 95% and later increased up to 100% at a temperature of 1200 °C. They also found that the equilibrium conversion of CO_2_ decreased as the temperature was increased from 300 °C (573 K) to 600 °C (873 K). This was due to the reaction of CO_2_ with two moles of H_2_ (Equation 1) to produce a large amount of carbon deposits and water since the reaction is exothermic and favorable at lower temperature.

[1]
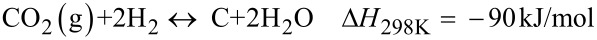


However, the equilibrium conversion of CO_2_ increased as the temperature was increased to above 600 °C (873 K), as Equation 2 and Equation 3 are favorable at high temperature. The results obtained for the equilibrium conversion of CH_4_ and CO_2_ showed that the ratio of unity was the best ratio to obtain a balanced conversion between CH_4_ and CO_2_. As the ratio of CO_2_/CH_4_ increased, CH_4_ became the limiting reactant and CO_2_ was the excess reactant, so the limiting reactant was consumed more and the consumption of excess reactant was not completed.

[2]
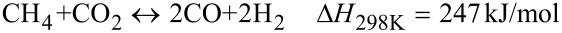


[3]
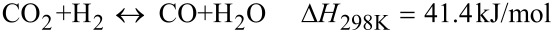


In terms of catalytic performance, pressure is one of the factors which influences the activity of the catalyst in a DRM reaction because as the pressure increases, carbon deposition increases significantly. In addition, according to Chein et al. [13], the carbon deposit could increase to approximately 50% from atmospheric to 10 bar. It has been reported that as the conversion of reactants (CH_4_ and CO_2_) decreased, the yield of H_2_ and CO also decreased and the carbon deposition increased with an increase in pressure, which indicates that the DRM reaction is not favored at high pressure [13,59–60]. This is probability because the collision of gas molecule and the active site surface of the catalyst increases and the surface concentration along with residence time for the CH*_x_* species also increase as the pressure increases. The increase in pressure leads to a synchronization of carbon deposition and consumption whereby the rate of carbon deposition is higher than the consumption rate, resulting in catalyst deactivation due to carbon deposition [61].

In the DRM reaction, the carbon deposition and side reactions, especially the RWGS reaction, are known as a major problem to the stability of the catalyst and prevent a high yield of syngas production (H_2_ and CO) [62]. Jafarbegloo et al. [63] also reported that the mole fraction of H_2_O increased as the pressure in the DRM reaction was increased. Even at atmospheric pressure, the formation of H_2_O was observed which indicates the occurrence of the RWGS reaction. This shows that the RWGS in the DRM reaction can be reduced but it cannot be eliminated according to thermodynamic equilibrium.

#### Role of inert gas in DRM

**Figure 1:**
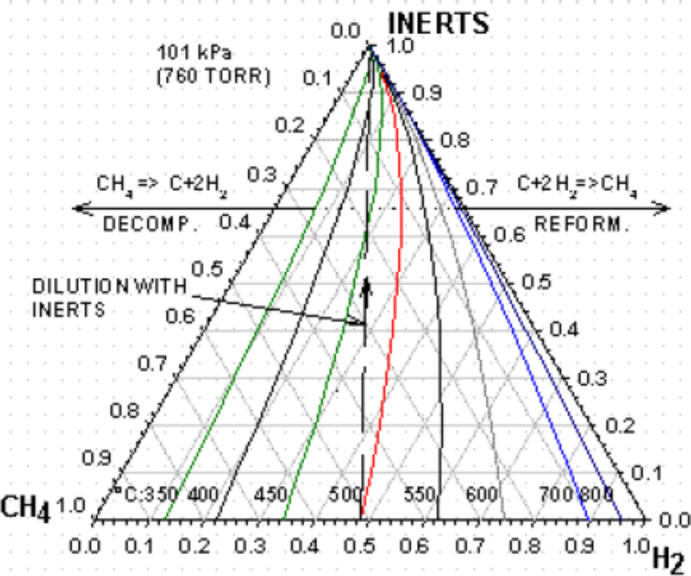
Methane decomposition equilibrium in the presence of inert gas at different temperatures. Reprinted with permission from [64], copyright 2002 Taylor and Francis Group.

The use of inert gases such as N_2_ can affect the DRM reaction especially in methane cracking (Equation 4). Figure 1 shows the relationship between CH_4_, H_2_, inert gas, and temperature in a ternary composition diagram [64]. The dotted line represents the presence of inert gas in the equilibrium mixture of CH_4_ and H_2_ at 500 °C. As the mole fraction of the inert gas increases, the reaction will increasingly favor thermodynamic CH_4_ decomposition. Furthermore, it can be said that at higher temperature, the inert gas does not affect the DRM reaction in terms of carbon deposition, so the effect of inert gas at high temperature could be negligible.

[4]
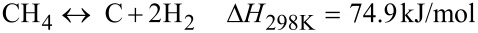


### DRM reaction mechanism

DRM is an endothermic reaction favored at high temperature and a low pressure. The reaction mechanism is extremely important for understanding the involved processes or for determining a solution to overcome catalyst deactivation. In general, the reaction mechanism depends on the choice of catalyst and all reactions involved in the DRM process. The overall proposed mechanism of the DRM reaction in the literature [57,65–66] follows three main steps: i) adsorption of CH*_x_* (*x* = 0–4) and H, and decomposition of CH_4_, ii) adsorption and activation of CO_2_ and iii) oxidation of CH and C.

The role of the various catalyst constituents is important for the adsorption of reactants onto the catalyst surface and for the weakening of the chemical bonds of the reactant for the dissociation process. In one proposal, CH_4_ activation was found to take place on the active sites. Niu et al. [66] proposed the mechanism of DRM over a Pt catalyst as shown in Equation 5. Equation 5 shows the adsorption of methane on the metal catalyst followed by dehydrogenation to produce hydrogen and a hydrocarbon species CH*_x_* (*x* = 0–3). When *x* = 0, carbon deposition occurs on the metal surface. Equation 6 describes the dissociation of CO_2_ into CO and O. The produced atomic H from the dehydrogenation process activates CO_2_ to form COOH before decomposition into CO and OH, as displayed in Equation 7. The H adsorbed by the catalyst surface activates CO_2_ to form HCOO*, which then dissociates into CHO* and H* (Equation 8). Then, in the following step (Equation 9), CH* is oxidized to produce CHO*/COH* and subsequently decomposes to CO* and H*. Equation 10 depicts the oxidation of CH* by OH*, becoming CHOH* as an intermediate before dissociating into CHO*/COH* and H*. Finally, Equation 11 shows the oxidation of C* by O* and OH*, resulting in CO* and COH*, respectively. The large majority of the adsorbed hydrogen species are then recombined to produce the hydrogen molecules that subsequently desorb into the gaseous phase. In the following equations, the symbol (*) indicates the surface of the catalyst active site in the reactions.

[5]
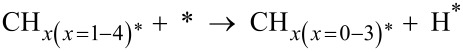


[6]
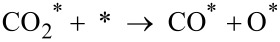


[7]
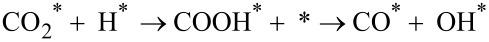


[8]
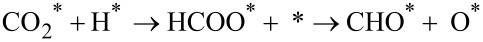


[9]
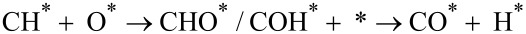


[10]
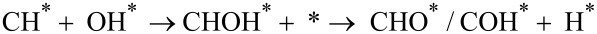


[11]
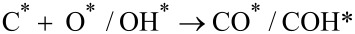


Another reaction mechanism, in which the activation of CO_2_ takes place on the surface of the support rather than the metal active site, was proposed by Fan et al. [65] for catalysts with basic supports, such as MgO. In this mechanism, CO_2_ is adsorbed on the catalyst support in the vicinity of the metal particles, CO_2_ (g) ↔ CO_2_ (support), to form carbonate species, CO_2_ (support) + O^2−^ ↔ CO_3_^2−^ (support). Then, the carbonate was reduced by the adsorbed hydrogen (CO_3_^2−^ (support) + 2H ↔ HCO_2_^−^ (support) + OH^−^) to form CO [65,67–68]. When Mg or MgO are added as promoters, CO_2_ is supposedly adsorbed onto the promoter and dissociated into CO and O (Equation 4). Then, the surface reaction occurs between the adsorbed oxygen on the promoter followed by deposition of carbon from CH_4_ decomposition on the catalyst active sites to form CO(O* + C* → CO*).

### Mechanism of carbon formation/removal in DRM

Catalyst deactivation is usually influenced by metal sintering and carbon deposition. Metal sintering may be caused by exothermic reactions and/or local overheating [69]. Carbon deposition is the major problem in the DRM; carbon deposition originates from reactions such as CH_4_ decomposition (Equation 4), the Boudouard reaction (Equation 12), and CO reduction reaction (Equation 13).

[12]
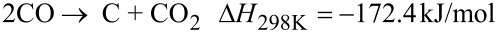


[13]
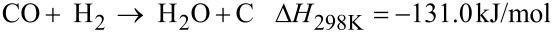


**Figure 2:**
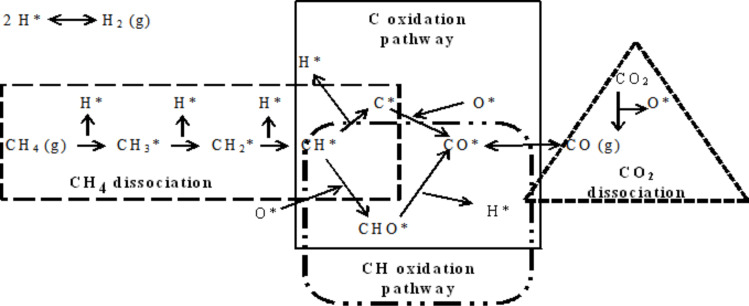
A schematic diagram of the DRM reaction on Ni metal. Adapted from [73].

Several studies have attempted to discover the phenomena of carbon deposition in terms of its formation and variations in chemical structure with the applied catalysts, feed gas ratio, and other process conditions [24,68,70–76]. In the past decade, most researchers have focused on catalyst development to decrease or eliminate carbon formation and have struggled to understand the mechanism of carbon formation. Different mechanisms according to reactant activation and type of applied catalyst have been proposed for the growth of carbon on the catalyst surface. Lobo et al. [77] suggested the basic idea for the mechanism of carbon formation based on three main steps: i) carbon formation from the surface reaction occurring from the decomposition of hydrocarbons on the metal surface; (ii) dispersion of carbon atoms through the bulk of active metal; and (iii) the progression of the deposited carbon. Wang et al. [73] proposed conducting the DRM reaction over Ni metal through four main pathways, namely, CH_4_ dissociation, CO_2_ dissociation, C oxidation, and CH oxidation, as shown in Figure 2. Interestingly, carbon formed only through the C oxidation pathway; meanwhile, CH was transformed to CO through CHO in the CH oxidation pathway, which bypasses the formation of carbon from CH_4_. Furthermore, carbon formation from methane dissociation could decrease if the rate of CH oxidation exceeded that of C oxidation. In contrast, carbon formed from the CO_2_ dissociation pathway could be related to the energy barrier in CO. First, CO_2_ dissociates on the catalyst into CO and O. Then, CO dissociates into C and O and forming carbon. Thus, the high energy barrier of CO could prevent the formation of carbon.

Djaidja et al. [78] highlighted that the inhibition of carbon deposition over a Ni–M/MgO catalyst is a result of increasing the adsorption of CO_2_ on the support surface, enhancing the rate of surface reaction and decreasing the rate of methane decomposition. Meanwhile, Theofanidis et al. [28] stated that carbon deposition could be solved with a higher distribution of active metal on the support surface, high basicity of catalyst surface to capture acidic CO_2_, and the addition of resources that offer an oxygen atom through their redox properties.

**Figure 3:**
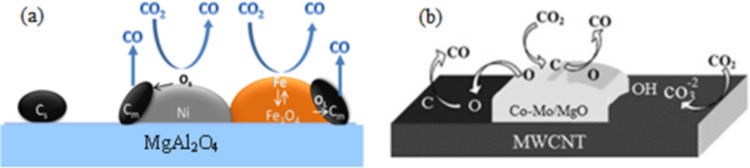
Schematic diagram of carbon species removal by CO_2_ over (a) Fe–Ni catalyst [28] and (b) Co–Mo–MgO/MWCNT nanocomposite. [69]. (C_s_: deposited carbon far from metals, O_s_: surface oxygen, O_L_: lattice oxygen, C_m_: carbon deposited on metals). Reprinted with permissions from [28] and [69], copyright 2016 and 2014, respectively, Elsevier.

Theofanidis et al. [28] proposed a mechanism for carbon removal using Fe–Ni/MgAl_2_O_4_ in a DRM reaction. They focused on the mechanism of carbon species removal by CO_2_. They proposed that the carbon removal mechanism involved two parallel processes, as clearly depicted in Figure 3a. First, CO_2_ was dissociated over Ni metal followed with the surface oxygen (O_s_) originating from O_2_ dissociation or by direct interaction with gas phase O_2_. Then, O_s_ combined with the carbon deposited on the Ni metal (C_m_) to form CO. The second process commenced with the oxidation of Fe by CO_2_ to form iron oxide (Fe_3_O_4_). Next, the lattice oxygen (O_L_) obtained from the reduction of Fe_3_O_4_ was pooled with carbon deposited on the metal (C_m_) to form CO. However, carbon located far from the active sites (C_s_) does not directly intermingle with CO_2_. Based on the results of this study, the application of MgAl_2_O_4_ as support could help the adsorption of more CO_2_ on the catalyst surface because of its alkaline property and consequently increase the rate of elimination of carbon deposited on the catalyst as a result of the dehydrogenation of CH_4_. Figure 3b exhibits the proposed mechanisms of carbon removal by Khavarian et al. [69], who demonstrated carbon removal by active surface oxygen produced from CO_2_ dissociation over active metal particles supported by multiwalled carbon nanotubes (MWCNTs). Interestingly, the active sites of the catalyst were regenerated, and stable conversions were accomplished. In brief, development of a reliable surface reaction mechanism is a complex process. However, there are various elementary mechanisms developed for the DRM reaction, which should be measured in the absence of heat and mass transfer limitations [29].

### Kinetic study in DRM

Several research studies have investigated how to inhibit or remove carbon formation at the early stages. It is believed that the development of a coke-resistant catalyst may lie in a detailed understanding of catalytic processes and kinetic studies. The reaction kinetics in the DRM depends on the type of catalysts and the involvement of reactants in the reaction. Even though the type of catalyst, the nature of the support and the reaction temperature range affect the reaction kinetics in DRM, there is still no available universal expression for the reaction kinetics in this process. Many studies have been carried out especially on developing elementary and microkinetic analyses for the understanding of reactions that occur in DRM [79–82]. Microkinetic analysis is an investigation based on elementary chemical reactions on the catalytic surface and their relationship with each other and with the surface during a catalytic cycle. Commonly, the kinetic parameters involved in reactions are extracted from experimental and theoretical studies using several methods, which include adsorption, surface reaction and desorption [82].

**Figure 4:**
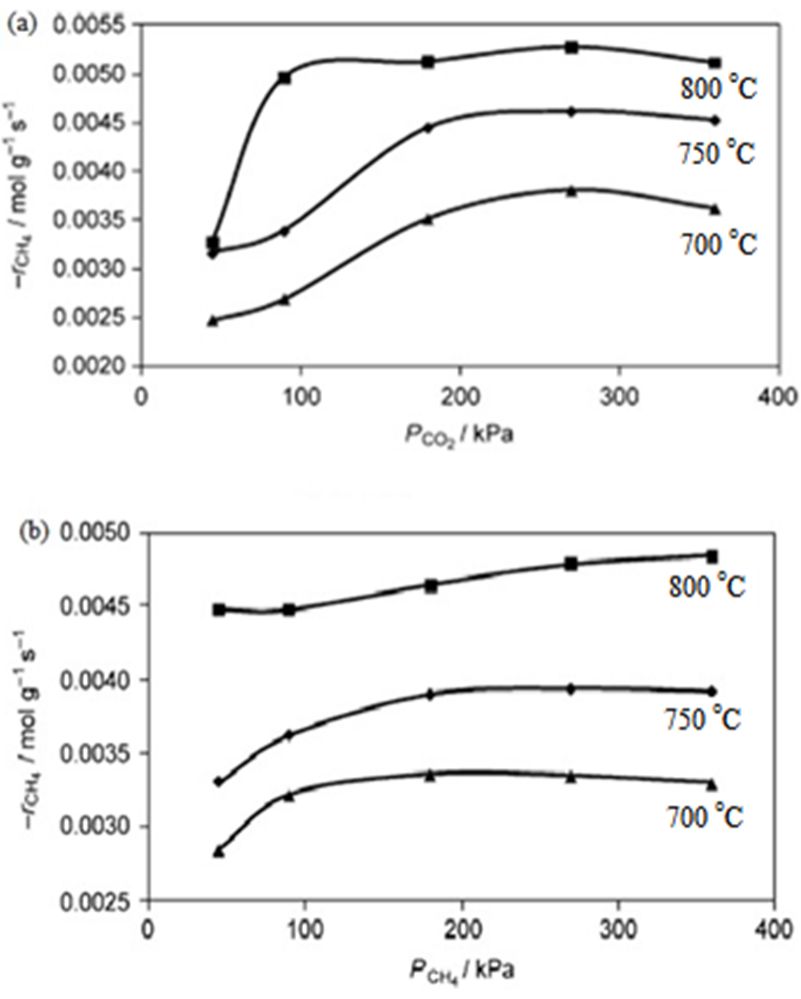
The partial pressure effect of (a) CO_2_ and (b) CH_4_ on the rate of CH_4_ reforming; *P*_CO2_ = 90 kPa. Reprinted with permission from [3], copyright 2011 Elsevier.

Fan et al. [3] performed a kinetic study over Ni–Co/MgO–ZrO_2_ at various partial pressures (45–360 kPa) and temperatures (700–800 °C). In this study, the effects of CH_4_ and CO_2_ partial pressure on the reforming rate were observed respectively. The results obtained emphasized that the partial pressure of CO_2_ showed a positive effect on the reforming rate compared to CH_4_, as shown clearly in Figure 4a,b. The authors declared that this is due to more CO_2_ being attracted and adsorbed to the surface of the catalyst, which has a higher basicity support and strong metal-support interaction compared to CH_4_.

Table 1 displays a comparison of the activation energies found in the literature using various catalysts for DRM [3]. The CO_2_ activation energy in this study recorded a lower value, which is 48.1 kJ/mol compared to that of CH_4_ and CO_2_ of previous studies. This is might be due to the existence of Mg, which is known to be a strong Lewis base, making it easier for CO_2_ to activate on the Ni–Co/MgO–ZrO_2_ catalyst.

**Table 1:** Activation energies of various catalysts for DRM [3].

Catalyst	*T* (°C)	Activation energy of CH_4_ (kJ/mol)	Activation energy of CO_2_ (kJ/mol)	Ref.

Ni–Co/MgO–ZrO_2_	700–800	52.9	48.1	[3]
Ni/Al_2_O_3_	500–700	50.9	56.1	[83]
Ni/MgO	450–550	92.1	87.9	[84]
Ni/CaO–Al_2_O_3_	620–690	106.8	98.8	[85]

**Figure 5:**
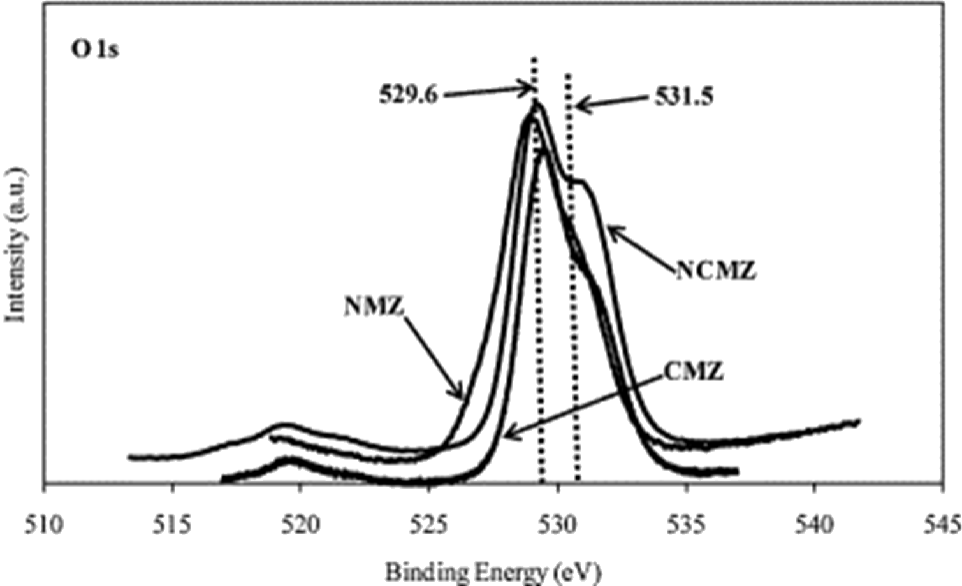
XPS spectra of O 1s of the catalysts. (NCMZ = Ni–Co/MgO–ZrO_2_, NMZ = Ni/MgO–ZrO_2_, CMZ = Co/MgO–ZrO_2_). Reprinted with permission from [52], copyright 2010 Elsevier.

The effect of the presence of MgO in maintaining the stability of the catalyst was clearly exhibited in the X-ray photoelectron spectroscopy (XPS) results, as shown in Figure 5. Based on a study by Fan et al. [52], Figure 5 shows a high binding energy of 531.5 eV. This indicates the high intensity of the hydroxyl group by the Ni–Co/MgO–ZrO_2_ catalyst, which reflects high surface basicity.

**Figure 6:**
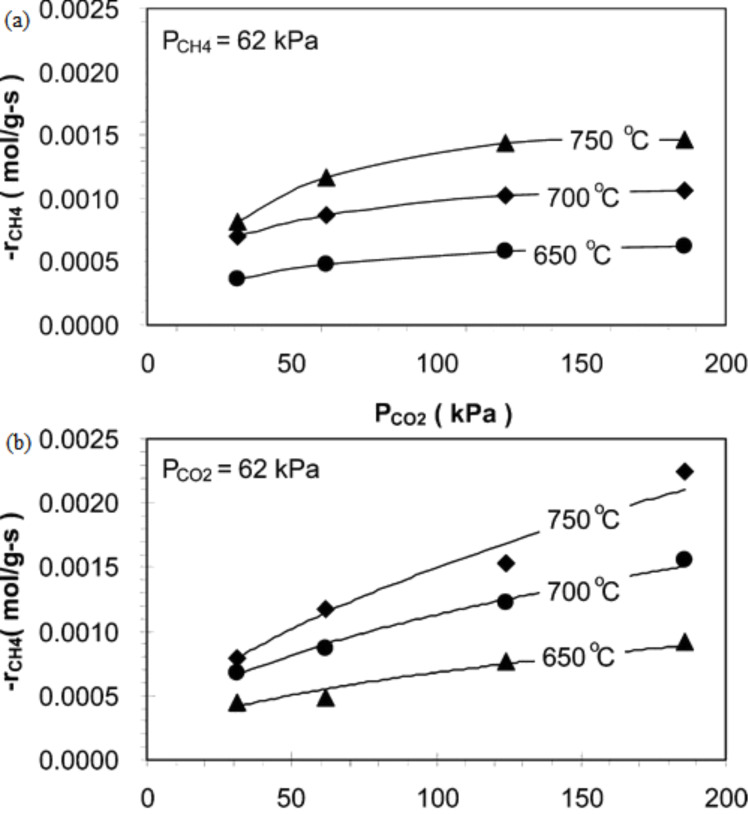
The reforming rates of DRM over the Ni–Co/Al–Mg-O catalyst affected by the (a) CO_2_ partial pressure and (b) CH_4_ partial pressure at different temperatures. Reprinted from [86], copyright 2009 American Chemical Society.

Kinetic studies of DRM over Ni–Co/Al–Mg-O have been carried out by Zhang et al. [86]. From this study, it was found that the rate of reforming is more prone to CH_4_ partial pressure rather than CO_2_ partial pressure, which is clearly displayed in Figure 6a,b. The authors mention that this might be due to a high amount of CH_4_ being adsorbed to the catalyst surface compared to CO_2_ at higher partial pressure.

**Figure 7:**
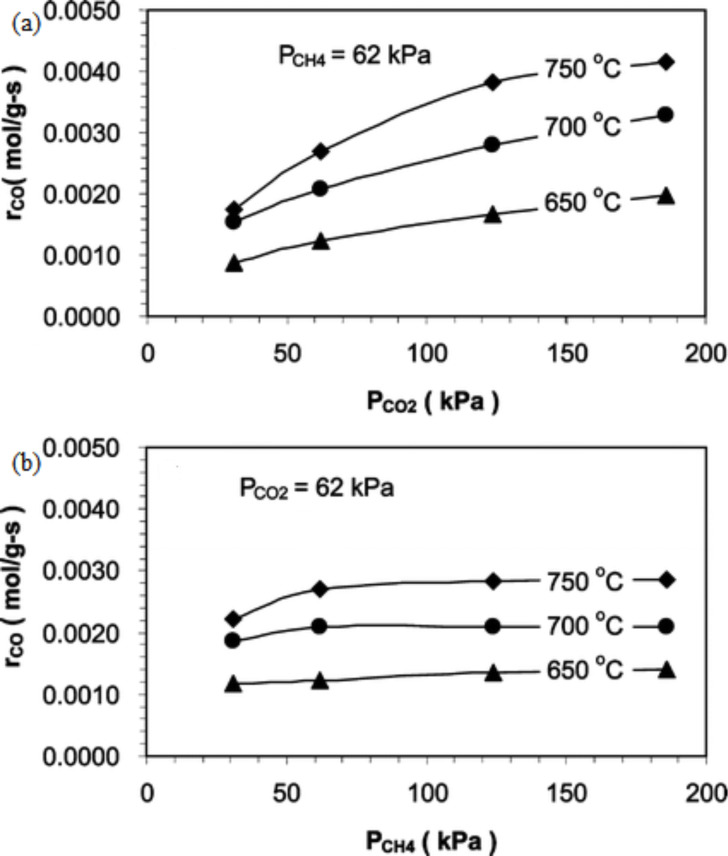
The formation rates of CO affected by (a) CH_4_ partial pressure and (b) CO_2_ partial pressure at different temperatures. Reprinted from [86], copyright 2009 American Chemical Society.

**Figure 8:**
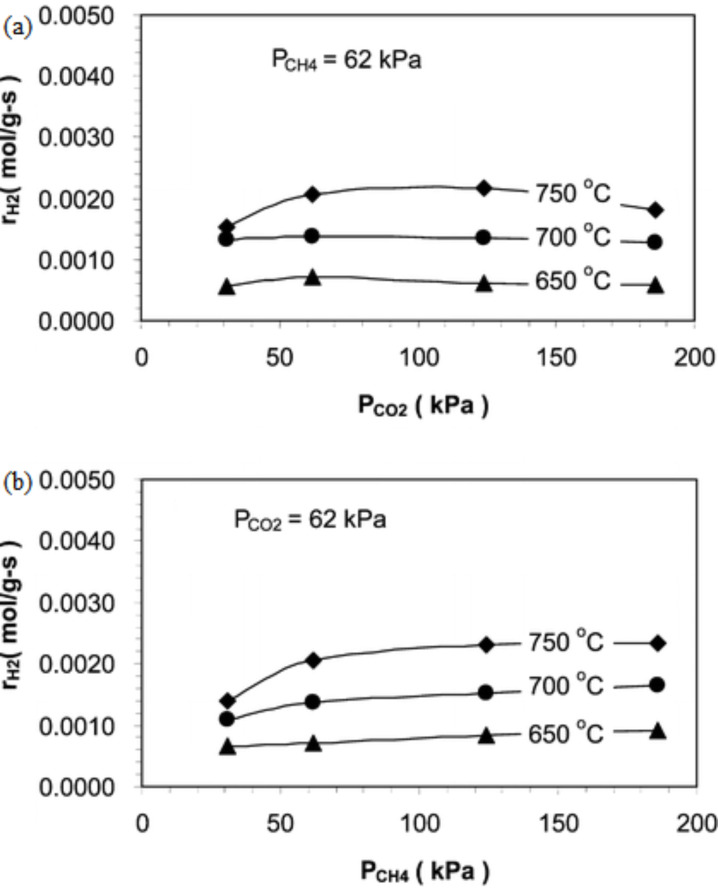
The formation rates of H_2_ affected by (a) CH_4_ partial pressure and (b) CO_2_ partial pressure at different temperatures. Reprinted from [86], copyright 2009 American Chemical Society.

Zhang et al. [86] also studied the formation rate of products influenced by the partial pressure of the reactants. Figure 7a,b shows that the rate of CO formation is more sensitive towards CO_2_ partial pressure compared to CH_4_ partial pressure. This could be attributed to the reverse water–gas shift (RWGS) reaction. Moreover, the H_2_ formation is relatively stable in both CO_2_ partial pressure and CH_4_ partial pressure due to the simultaneously occurrence of DRM and RWGS reactions. Figure 8a,b displays the effect of reactant partial pressure on H_2_ formation rates.

Table 2 outlines the values of activation energy for CO_2_, CH_4_, H_2_ and CO_2_ for various catalysts. The authors highlighted that the lowest activation energies were recorded by CO_2_. This is probably due to the existence of MgO, which is a strong Lewis base that acts as a support in this study, and can also activate CO_2_. The activation energy of H_2_ is greater than CO because of the occurrence of the RWGS reaction.

**Table 2:** Activation energies of various catalysts [86].

Catalyst	*T* (°C)	Activation energy of CH_4_ (kJ/mol)	Activation energy of CO_2_ (kJ/mol)	Activation energy of CO (kJ/mol)	Activation energy of H_2_ (kJ/mol)	Ref.

Ni–Co/Al–Mg–O	650–750	69.4	25.9	61.8	85.1	[86]
Ni/Al_2_O_3_	500–700	50.9	56.1	80.5	–	[83]
Ni/CaO–Al_2_O_3_	620–690	106.8	98.8	103	147.4	[85]

### Development of Mg-containing oxide catalysts

The use of Mg or MgO, as either a promoter or a support in catalyst components, has attracted considerable interest [50–52,78]. Catalyst supports and promoters perform different roles in chemical reactions. Catalyst supports are normally applied to increase the catalyst surface area for active metal dispersion and to enhance the properties of the catalyst [65,87–88]. However, supports sometimes act as a transportation medium for the active sites while remaining catalytically inactive during the reaction [65]. Park and Song [89] stated that conventional supports could stabilize active metal and prevent the occurrence of leaching or metal agglomeration. The authors further mention that supports can not only transport the reactants to the reaction center, but can also control diffusion rates of reactants and products and donate protons or electrons to alter total reactivity. Promoters can be classified as small amounts of additives to active metals or support. The promoter of supports is used to prevent unwanted catalyst activity, such as carbon deposition, while promoters are added to active components to enhance reaction activity [90].

The activity and stability of catalysts prepared by various preparation methods have been observed by numerous researchers [91–94]. The catalyst preparation technique is an alternative approach to achieve carbon inhibition by controlling particle size and metal loading during catalyst preparation. The pivotal factors determining the activity and the selectivity of the catalysts are the dispersion and the physico-chemical characteristics of active metals on the support catalyst. Three general approaches are applied for the catalyst preparation, including (i) deposition of active metals onto a support; (ii) precipitation of materials with significant properties; and (iii) preparation of compact, nonporous compounds, or alloys with an active component, followed by the extraction of inactive particles, leaving a porous, high-surface-area active phase [95]. Arora [96] further highlighted that strong metal–support interactions were observed at low metal loadings (<12%), and a small particle size (<6 nm) could prevent carbon accumulation on the catalyst surface. The effects of operating conditions were investigated by adjusting the reaction temperature, feed ratio, space velocity, and temperature. The following sections investigate the influence of these variables on catalytic activity and morphology of deposited carbon. The target product compositions are also outlined.

#### Application of MgO as a support

Djaidja et al. [78] prepared a Ni/MgO catalyst via an impregnation method to test the performance in a DRM reaction. The conversion of CO_2_ and CH_4_ with MgO using 5 mol % of Ni loading were 98.1% and 97.1%, respectively, and a H_2_/CO ratio of 1.1 was recorded with a low quantity of deposited carbon. Notably, a slightly higher coke deposition was recorded in the catalyst without Mg. Thus, this finding proves that coke could be minimized with an increase in MgO concentration in the catalyst because MgO exhibits alkaline characteristics. Its high basicity and similar crystal structure with NiO render MgO as highly suitable for Ni-based catalyst supports. Strong metal–support interaction between MgO and NiO formed a basic solid solution (Ni*_x_*Mg_(1−_*_x_*_)_O) and could consequently enhance CO_2_ adsorption and Ni particle size. Moreover, the reduced Ni/MgO catalysts may result in a high distribution of Ni particles and may prevent the occurrence of particle sintering during DRM reactions. Therefore, this catalyst exhibits the potential for long-term reaction and resistance to carbon formation. As highlighted, 15 wt % of Ni metal loading is required for substantial metal–support interaction and high catalytic activity in DRM [97].

Hua et al. [36] synthesized a Ni/MgO catalyst via a dielectric barrier discharge plasma and compared the catalyst with the ones prepared via the conventional impregnation method. The Ni/MgO catalyst prepared using a dielectric barrier discharge plasma presented a smaller particle size, higher dispersion, and higher specific surface area. Moreover, the recorded CO_2_ and CH_4_ conversions were 20% higher than that of the conventionally prepared catalyst. The plasma treatment method is suggested to be more appropriate for the preparation of Ni/MgO, offering high activity and stability, and a lesser amount of deposited carbon on the catalyst compared to using the Ni/MgO catalyst prepared via impregnation. Zanganeh et al. [34] used the co-precipitation method to prepare a NiO–MgO nanocrystalline solid solution catalyst. High Ni (25 wt %) content negatively affected the supression of carbon deposition on the solid solution catalyst. However, high Ni dispersion and the basicity of the support surface could inhibit carbon formation in DRM on the reduced Ni_0.03_Mg_0.97_O solid solution catalyst.

DBD plasma, also known as silent discharge, is a conventional cold plasma phenomenon that has been widely applied in the preparation of multi-oxide catalysts. This method can overcome the problem that arises from the use of thermal calcination techniques at high temperature. This plasma treatment is the most well-known technique because of its requirement for a low operation temperature with greatly energetic electrons for catalyst preparation. Thus, this method is very friendly towards heat sensitive substrates. Rapid reactions occur between the active plasma species and catalyst precursors compared to the thermal decomposition technique. Therefore, this may encourage the rapid nucleation of the crystals under plasma treatment. The application of low temperature may result in slow crystal growth, which contributes to the production of a catalyst with a small particle size or high metal dispersion. Furthermore, plasma treatment has been proven to show superb performance in surface treatment and modification. Moreover, this technique is able to efficiently decompose precursors at low temperature. Greater dispersion of oxide nanoparticles on another oxide support can be obtained via plasma treatment without any additional chemicals. [98]

According to previous studies [36,99–100], plasma-treated catalysts have a smaller particle size, enhanced metal dispersion and increased specific surface area. Moreover, Yan et al. [101] confirmed that plasma treated catalysts can improve the interaction between Ni and the SiO_2_ support and produces less defect sites on the Ni particles. The authors further remarked that this plasma treatment could suppress inactive carbon deposition and enhance catalyst performance. Yan et. al [102] studied the preparation methods of the Ni catalyst, which are the DBD plasma and thermal decomposition. It is interesting to note that plasma treatment produces more Ni (111) surface with fewer defects, which is most suitable for suppressing carbon deposition. Meanwhile, the structure for Ni catalysts were treated via thermal decomposition recorded with a great number of defect sites, which consist of Ni (100) and Ni (101) surfaces. These surfaces could increase carbon deposition rate. Table 3 summarizes the development and process conditions of Mg-containing oxide catalysts used in DRM.

**Table 3:** Literature summary on the development and process conditions of Mg-containing oxide catalysts in DRM. PM: Preparation method; W: metal loading; CC: calcination conditions; RC: reaction conditions; SV: space velocity.

Catalyst	PM	W (wt %)	CC	RC	Conversion				

					CH_4_ (%)	CO_2_ (%)	H_2_/CO	Carbon	Ref.

Ni–Ce/Mg–Al	carbonate co- precipitation	Ni/Mg: 2/1^a^	*T* = 500 °C, *t =* 16 h	*T* = 500– 800 °C, CH_4_/CO_2_ 1:1^a^, SV = 30 L/h·g	90	95	1.2– 1.5	–	[50]
Ni/MgO	impregnation	Ni: 5–10 wt %	*T* = 600– 900 °C	*T* = 800 °C, CH_4_/CO_2_ 1:1^a^	97.15	98.1	1.1	–	[78]
Ni/MgO	dielectric barrier discharge plasma	Ni: 10 wt %	*T* = 700 °C, *t =* 4 h	*P =* 1 atm, *T* = 700 °C, CH_4_/CO_2_/NO_2_ 1:1:2^a^, SV = 96 L/h·g	46	52	0.84	3.9 wt %	[36]
Pt–Ni–Mg/ Ceria–zirconia	co- precipitation; incipient wetness impregnation	Pt/Ni/Mg: (0.2–2)/8/8 wt %	*T* = 800 °C, *t =* 4 h	*P =* 1 atm, *T* = 430– 470 °C, CH_4_/CO_2_ 1:1^a^, SV = 68,000 h^−1^	≈10	≈20	0.23	no coke deposited	[51]
Ni–Co/ MgO–ZrO_2_	surfactant- assisted impregnation; impregnation	Ni: 3, Co: 3 wt %	*T* = 800 °C, *t =* 3 h	*P* = 1 atm, *T* = 750 °C, CH_4_/CO_2_ 1:1^a^, SV = 125 L/h·g	80	84	0.97	0.89 mg_C_/g_catalyst_	[52]
Ni/ZrO_2_–MgO	co- precipitation; wet impregnation	MgO: 1–5 wt %	*T* = 700 °C, *t =* 3 h, rate = 5 °C/min	*P =* 1 atm, *T* = 600 °C, CH_4_/CO_2_ 1:1^a^	30	32	–	0.26–0.31 mg_C_/mg_catalyst_	[103]
Ni–Mg/Al_2_O_3_	reverse microemulsion	Ni/Mg: 10^a^	*T* = 800 °C, *t =* 2 h, rate = 10 °C/min	*P =* 1 atm, *T* = 400– 700 °C, CH_4_/CO_2_/He 20:20:60^a^, SV = 6,000 h^−1^	58	69	0.67	0.5 wt %	[19]
Ni/Al_2_0_3_–MgO	sol–gel	Ni: 10 wt %	*T* = 600 °C, *t =* 5 h	*P =* 1 atm, *T* = 550– 850 °C, CH_4_/CO_2_ 0.5–2.0^a^, SV = 24– 60 L/h·g	93– 95	97– 99	0.5– 0.98	–	[37]
Ni/Al_2_0_3_–MgO	impregnation	Ni: 10 wt %	*T* = 500 °C, *t =* 2 h	*P =* 1 atm, *T* = 550– 850 °C, CH_4_/CO_2_ 0.5–2.0^a^, SV = 24– 60 L/h·g	83– 85	85– 87	0.38– 0.78	–	[37]
Ni–MgO–Al_2_O_3_	sol–gel	Ni: 15 wt %, MgO/(MgO+Al_2_O_3_) 0–1^a^ optimum ratio 0.44–0.86^a^	*T* = 750 °C, *t =* 5 h	*P =* 1 atm, *T* = 800 °C, *t =* 40 h, CH_4_:CO_2_:N_2_ 1:1:1^a^, SV = 36 L_CH4_/h·g	83.6– 90	88.8– 92	–	13 wt %	[104]
Ni–MgO–Al_2_O_3_	co- precipitation	Ni: 15 wt %, MgO/(MgO+Al_2_O_3_) 0–1^a^ optimum ratio 0.44–0.86^a^	*T* = 750 °C, *t =* 5 h	*P =* 1 atm, *T* = 800 °C, *t =* 40 h, CH_4_:CO_2_:N_2_ 1:1:1^a^, SV = 36 L_CH4_/h·g	83.6	88.8	–	–	[104]
Ni–Mg/SiO_2_	impregnation	Ni: 10 wt %, Mg: 5 wt %	*T* = 800 °C, *t =* 5 h	*P =* 1 atm, *T* = 800 °C, *t =* 10 h, CH_4_/CO_2_ 1:1^a^	74.6	78.6	1.49	6.56 wt %	[105]
Ni/MgO/γ-Al_2_O_3_	cold plasma	Ni: 12 wt %, MgO: 2 wt %	*T* = 550 °C, *t =* 4 h	*P =* 1 atm, *T* = 800 °C, *t =* 400 h, CH_4_/CO_2_ 4:6^a^, SV = 30 L/h·g	78– 97	74	–	5 wt %	[106]
NiO/MgO	impregnation	NiO/MgO 20:100^b^	*T* = 600– 800 °C, *t =* 1.5 h	*P =* 0.1 MPa, *T* = 800 °C, *t =* 5 h, CH_4_/CO_2_ 1:1^a^, SV = 20 L/h·g	85– 92	90– 94	–	–	[94]
Ni–Mg–Al	co- precipitation	Ni/Mg 1–5^a^	*T* = 400– 800 °C, *t =* 6 h	*P =* 1 atm, *T* = 700 °C, CH_4_/CO_2_/N_2_ 1:2:9^a^, SV = 45 L/h·g	95– 96	49– 50	–	2.8–3.7 wt %	[92]
Ni/MgO	wet impregnation	Ni: 8.8 wt %	*T* = 400– 950 °C, *t =* 5 h	*P =* 0.1 MPa, *T* = 750 °C, CH_4_/CO_2_ 1:1^a^, SV = 16– 240 L/h·g	84	–	–	–	[23]
La–NiMgAl	co- precipitation	Ni: 2.1 wt % Mg/Al 1.7^a^	*T* = 250– 750 °C, *t =* 2 h	*T* = 700 °C, *t =* 50 h, CH_4_/CO_2_ 1:1^a^	25– 31	32.5– 55	–	0–0.06 g_C_/g_catalyst_·h	[107]
NiCoMg/Al_2_O_3_	co- impregnation	Ni: 3, Co: 3, Mg: 3 wt %	*T* = 500 °C, *t =* 5 h	*P =* 1 atm, *T* = 850 °C, *t =* 3000 h, CH_4_/CO_2_/N_2_ 1:1:1^a^, SV = 80,000 h^−1^	95.1	96.2	0.982	1.3 wt %	[54]
Ni/MgO	wetness impregnation	Ni: 5–15 wt % Optimum 15 wt %	*T* = 500 °C, *t =* 4 h	*P =* 1 atm, *T* = 500– 700 °C, *t =* 5 h, CH_4_/CO_2_ 2:1–1:2^a^, SV = 6– 24 L/h·g	25– 86.29	27– 86.77	0.64– 0.91	–	[108]
Ni/MgO (111)	impregnation	Ni: 2–20 wt %	*T* = 650 °C, *t =* 5 h	*P =* 1 atm, *T* = 450– 650 °C, *t =* 10 h, CH_4_/CO_2_ 1:1^a^, SV = 36 L/h·g	28– 60	40– 65	–	–	[109]
Ni–CeO_2_/MgO	surfactant- assisted precipitation	Ni: 5–15 wt % optimum 10 wt %	*T* = 500 °C, *t =* 4 h	*P =* 1 atm, *T* = 550– 700 °C, *t =* 20 h, CH_4_/CO_2_ 4:1–1:2^a^, SV = 6– 18 L/h·g	27– 95	30– 97	–	–	[110]
Ni/MgO	surfactant- assisted precipitation	Ni: 5–15 wt %, Mg/Al 1:1^a^ optimum 7 wt % Ni	*T* = 500 °C, *t =* 2 h	*P =* 1 atm, *T* = 550– 700 °C, *t =* 5 h, CH_4_/CO_2_ 2:1–1:3^a^, SV = 6– 24 L/h·g	67.5– 97.2	62.4– 97.4	0.58– 0.97	–	[111]
NiMgAl	sol–gel	Ni: 5 wt %, Mg/Al 5:1–1:5^a^	*T* = 600 °C, *t =* 4 h	*P =* 1 atm, *T* = 550– 700 °C, *t =* 5 h, CH_4_/CO_2_ 1:1^a^, SV = 12 L/h·g	55	80	1.2	–	[112]
Ni_0.5_Mg_2.5_Al_0.9_La_0.1_O_4.5_	co- precipitation	La: 0.04–0.15^a^	*T* = 500 °C, *t =* 16 h	*P =* 1 atm, *T* = 600– 700 °C, *t =* 13 h, CH_4_/CO_2_ 1:1^a^, SV = 7200 h^−1^	96	90	1.1	20.8 wt %	[113]

^a^molar ratio; ^b^weight ratio.

#### Application of MgO as a co-support

Abdollahifar et al. [49] investigated catalyst performance by applying the ultrasound-assisted impregnation (sonochemistry) method in the synthesis of a Ni/Al_2_O_3_–MgO catalyst for a DRM reaction. The measured surface area for the Ni/Al_2_O_3_–MgO catalyst was 53.25 m^2^/g. Ni nanoparticles with a diameter of 21.4 nm were dispersed on the support. For the catalyst performance, at a reaction temperature range of 750 °C to 800 °C was used and CO_2_ conversion and H_2_/CO ratios almost achieved thermodynamic equilibrium. At the reaction temperature range of 500 °C to 800 °C, the recorded CO_2_ is markedly higher than CH_4_ conversion and the H_2_/CO ratio reaches unity. The Ni/Al_2_O_3_–MgO catalyst was stable without deactivation for up to 53 h at a reaction temperature of 700 °C. The recorded performance might be influenced by the high surface area of Al_2_O_3_ and the high alkaline properties and thermal stability of MgO, which is more suitable for CO_2_ and CH_4_ adsorption. In fact, MgO offers low surface area, which could further be improved, and yields optimum texture properties with a combination of alumina [45,114–117].

Based on the investigation of the morphology, structure, and catalytic properties of the Ni–Ce/Mg–Al catalyst in DRM by Daza et al. [50], Ce and Mg are concluded to exert a synergistic effect on CO_2_ adsorption. Although the basic properties of Ce exert a significant effect on catalyst basicity, the bulk CeO_2_ possesses low basic properties. Therefore, the combination of Ce with Mg improves the catalyst basicity site. CeO_2_, commonly known for its oxygen storage capacity, contains a great concentration of highly mobile oxygen vacancies which could reduce the deposition of carbon on the catalyst surface [76]. In fact, CO_2_ molecules are more attracted to the base center (–Mg–OH group) and then dissociate on Ce_2_O_3_ via electron transfer to CO_2_ through oxygen vacancies to form CO_2_ and CeO_2_. Thus, the base center is most suitable for adsorbing the largest amount of CO_2_. The catalyst was stable for up to 50 h of reaction at 700 °C with an equal feed CO_2_/CH_4_ ratio. The conversion of CO_2_ and CH_4_ was in the range of 70 to 80% when the obtained H_2_/CO ratio was between 1 and 1.5.

The influence of the synthesis method on the Ni/Al_2_O_3_–MgO catalyst by sol–gel and impregnation techniques was investigated by Hassani Rad et al. [37]. A higher specific surface area (178 m^2^/g) was recorded with the MgO-doped catalyst obtained by the sol–gel method than that obtained using the impregnation method (70 m^2^/g). As a consequence, the preparation technique significantly affected the catalytic performance of the Ni/Al_2_O_3_–MgO catalyst and the Ni/Al_2_O_3_–MgO catalyst prepared using the sol–gel method, exhibiting large yields for H_2_, CO, and H_2_/CO, which were 90%, 98%, and 98%, respectively, at a reaction temperature of 850 °C. Evidently, the application of the sol–gel method and MgO as a support promoter improved the surface area and Ni dispersion and resulted in highly homogeneous morphology and small particle size but affected the adsorption of reactants on the catalyst. This research study is associated with the work of Sajjadi et al. [38], who used the sol–gel method to prepare a Ni–Co/Al_2_O_3_–MgO–ZrO_2_ catalyst. The findings showed that application of the sol–gel method with the addition of MgO as a support promoter produced considerable homogeneity of metal composition, small particle size, and enhanced the particle distribution and surface area.

In the study conducted by Min et al. [104], a comparative study was conducted between the sol–gel and co-precipitation methods for the preparation of a Ni–MgO–Al_2_O_3_ catalyst. The Ni particle size was uniformly distributed in the catalysts prepared via sol–gel. TGA results clearly showed that 13% and 7.7% carbon was deposited on the catalysts prepared via the sol–gel method and the co-precipitation method, respectively. Therefore, they concluded that the sol–gel method is preferable for Ni–MgO–Al_2_O_3_ catalyst preparation and possibly for inhibiting the formation of carbon. Xu et al. [106] prepared a Ni/MgO/γ-Al_2_O_3_ catalyst via the cold plasma technique and discovered an enhanced distribution of Ni particles. Moreover, the addition of MgO in this catalyst prevented the agglomeration of Ni particles and increased the basicity of the catalyst; consequently, more CO_2_ was adsorbed on the catalyst surface. The adsorption and activation of CO_2_ decreased the carbon deposition. Notably, the catalyst performance showed high stability for up to 400 h of reaction. Fan et al. [52] carried out DRM over a Ni–Co/MgO–ZrO_2_ catalyst synthesized by applying impregnation and surfactant-assisted impregnation methods. Reportedly, MgO stabilized *t*-ZrO_2_ and influenced the particle size. Furthermore, the addition of MgO to the ZrO_2_ support suppressed carbon deposition, which occurred between the crystallites of *t*-ZrO_2_ and prohibited a shift of the zirconia support from the tetragonal phase to the monoclinic phase. This catalyst tends to survive with low carbon formation (0.89 mg/g_catalyst_/h for 40 h with CO_2_ and CH_4_ conversion of 80% and 84%, respectively). García et al. [103] studied the effects of MgO on the basicity and performance of the Ni/ZrO_2_ catalyst in DRM. The catalyst was synthesized using two methods, wet impregnation and co-precipitation. A high carbonaceous residue (0.8 mg C/mg catalyst) was discovered on the catalyst surface with the absence of Mg in comparison to the amount of carbon deposition of 0.26 mg C/mg catalyst on the catalyst with an additional of 2.3% and 0.4% MgO. The conversion for CO_2_ and CH_4_ was 30% and 32%, respectively. The presence of MgO in the catalyst resulted in increased thermal stability in stabilizing the zirconia tetragonal phase, improved basicity sites for the support, and decreased reducibility of Ni^2+^.

Li et al. [118] designed a Ni@Ni–Mg phyllosilicate core–shell catalyst using the hydrothermal treatment of Ni@SiO_2_ with Mg(NO_3_)_2_. The alkalinity and porosity of the catalyst can be controlled during various hydrothermal treatment durations applied (such as 2.5, 10, and 24 h). Interestingly, the optimum hydrothermal treatment time for this catalyst was 10 h, resulting in good catalytic performance of 80% and 78% CO_2_ and CH_4_ conversion, respectively. Moreover, the increase in hydrothermal treatment time enhanced the basicity of the catalyst from the high exposure to the Mg phase. The strong basicity properties of Mg enhanced CO_2_ adsorption and suppressed carbon deposition via the reverse Boudouard reaction (C + CO_2_ ↔ 2CO). However, the core–shell structure became unstable when the catalyst was exposed to an excessively long treatment time.

**Figure 9:**
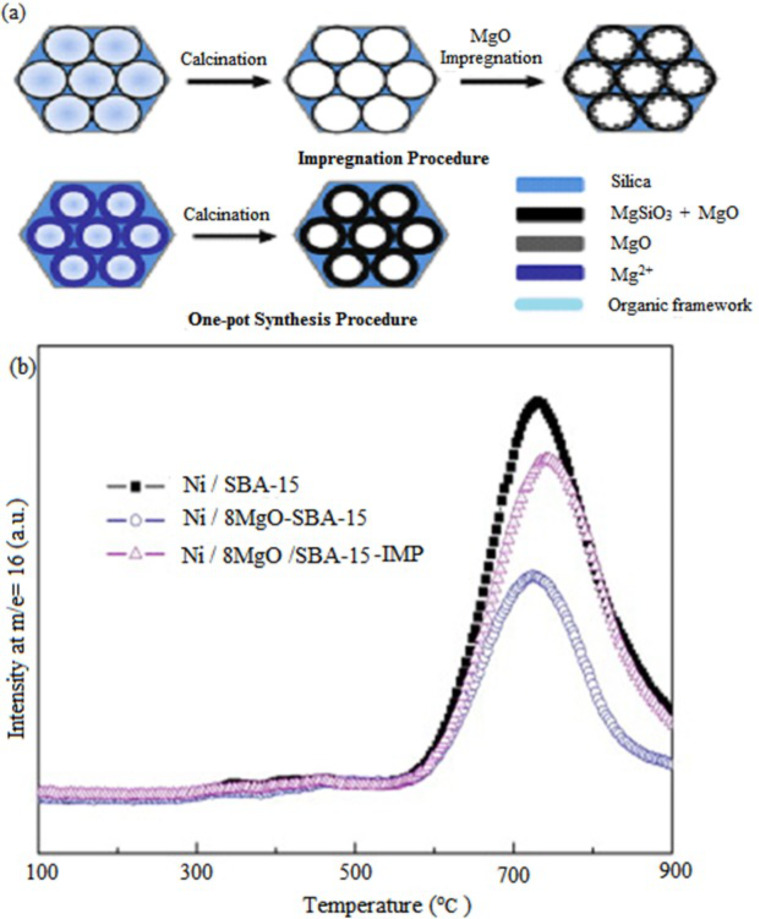
(a) Schematic illustration of the two synthesis methods for the MgO basic sites formation on SBA-15, (b) TPH profiles of spent catalysts after 40 h stability test. Reprinted with permission from [119], copyright 2013 Elsevier.

Wang et al. [119] prepared and analyzed a MgO-SBA-15 catalyst via two different methods, which are the one-pot synthesis method and impregnation method as shown in Figure 9a. As a result, the catalyst prepared using the one-pot synthesis showed a better order of MgO-coated sample in the mesostructure of SBA-15, a larger BET surface area, and formed more basic sites compared to the conventional impregnation method. The authors further impregnated the basic sites with nickel metal and tested it in DRM. The MgO-coated Ni/SBA-15 catalysts showed greater catalyst performance and stability compared to the Ni/SBA-15 sample. Figure 9b depicts the total petroleum hydrocarbons (TPH) profiles of spent catalyst after 40 h of stability. It is interesting to note that 8 wt % of MgO loading exhibited excellent catalytic activity. The SBA-15 supported catalyst showed that the confinement of pore walls inhibited the Ni particle aggregation. Meanwhile, the MgO coating showed a higher dispersion of Ni metal and highly basic sites than the MgO impregnated method. It was also found that this structure improved stability and could inhibit the formation of filamentous and encapsulating carbon. Deactivation of the catalyst occurs over the Ni/SBA-15 sample since a large amount of graphitic carbon species were formed.

#### Application of Mg as a promoter of active metal

Elsayed et al. [51] demonstrated DRM over Pt–Ni–Mg/ceria–zirconia synthesized via precipitation and the incipient wetness method. The largest amount of basic sites were found in the Ni–Mg/(Ce_0.6_Zr_0.4_)O_2_ catalyst, but the basicity site characteristics decreased with the addition of Pt to the catalyst. Consequently, the lowest CO_2_ and CH_4_ conversions were obtained and no carbon was deposited on the catalyst surface. Moreover, the catalyst was of a good composition, given that the DRM process could be performed within the low temperature range of 430 °C to 470 °C. The catalyst was stable for up to 100.5 h without carbon deposition.

García-Diéguez et al. [19] developed a Ni–Mg active metal material supported on alumina via the reverse microemulsion method and impregnation method in a comparative study. Mg was proven to stabilize Ni on the catalyst surface by preventing the diffusion of Ni particles into the alumina lattice, indirectly suppressing carbon deposition. Table 4 shows the average particle size of Ni and carbon content in the catalysts after the reaction. The NiMg/Al_2_O_3_ catalyst synthesized using the microemulsion method recorded a lower particle size (16 nm) and carbon content (0.5 wt %) compared with that of the NiMg/Al_2_O_3_ catalyst prepared via the impregnation method (20 nm for and 13 wt %, respectively). Based on the reactivity in DRM, both NiMg/Al_2_O_3_ catalysts prepared via different methods showed similar CO_2_ conversion of 27% at a reaction temperature of 600 °C, whereas the NiMg/Al_2_O_3_ catalyst prepared via the impregnation method showed a slightly higher CO_2_ conversion of 71% at a reaction temperature of 700 °C. The NiMg/Al_2_O_3_ catalyst synthesized via reverse microemulsion showed a CH_4_ conversion of 58%, which was higher than that of the catalyst prepared via other methods. The obtained ratios of H_2_/CO for the catalysts prepared using both methods were not markedly different, at 0.66–0.67. In terms of the preparation method, the reverse microemulsion method was preferable for the DRM process, owing to the good activity and stability of the catalyst, which minimized carbon deposition and caused considerable interaction between Ni and Mg.

**Table 4:** The average particle diameter, *D*_p_, of Ni^0^ and the carbon content in the catalysts after reaction [19]. ME = microemulsion method; IM = impregnation method. DRM = dry reforming of methane; DRP = dry reforming of propane.

Catalyst	DRM		DRP	

	*D*_p_ Ni^0^ (nm)^a^	C content (wt %)^b^	*D*_p_ Ni^0^ (nm)^a^	C content (wt %)^b^

NiMg/Al_2_O_3_ IM	20	13	25	26
NiMg/Al_2_O_3_ ME	16	0.5	16	5

^a^Calculated by the Scherrer equation; ^b^Obtained by elemental analysis.

Macedo Neto et al. [120] studied the synthesis of Ce in Ni–Mg–Al layered double hydroxides by co-precipitation using ultrasonication. Sonication significantly increased not only the specific surface area but also the incorporation of Ce in the structure. In fact, the ultrasonication method guarantees a uniform distribution of nanoparticles on the catalyst support without aggregation. Zhu et al. [105] introduced Mg as a promoter to the Ni/SiO_2_ catalyst synthesized via the impregnation method. The Ni–Mg/SiO_2_ catalyst was deactivated after a 30 h of reaction time due to the deposition of inert carbon, which is difficult to oxidize. However, the addition of Mg caused the reduction of the RWGS reaction; thus, a high H_2_ production was obtained.

Yan et al. [121] investigated the effects of the addition of MgO on the Ni catalyst in the DRM. In their investigation, a good dispersion of nickel oxide and MgO promoter was reported over a γ-Al_2_O_3_ support. In addition, the MgO promoter remarkably retarded the formation of the NiAl_2_O_4_ phase during the reaction. The addition of MgO to the catalyst in the presence of CeO_2_ inhibited self-dispersion and promoted Ni dispersion on the catalysts. Alipour et al. [68] reported that the addition of MgO to the Ni catalysts supported on nanocrystalline Al_2_O_3_ decreased the surface area of the catalysts. MgO has the most beneficial effect for catalytic activity and suppressed the carbon formation in comparison with other alkaline earth promoters such as CaO and BaO investigated in this work. In fact, adding MgO to the catalyst decreased the reduction temperature of the NiO species and increased the catalyst reducibility.

#### Influence of metal loading

Khajenoori et al. [110] conducted the DRM reaction over a Ni–CeO_2_/MgO catalyst with various Ni loadings (5–15 wt %) at a temperature of 550 °C to 700 °C. Thus, the CO_2_ and CH_4_ conversions showed significant increments when the Ni loading increased up to 10 wt %. However, the catalyst performance decreased with Ni loading greater than wt % because of the formation of large Ni crystals and, consequently, low dispersion. Moreover, the 10% Ni–7% CeO_2_/MgO catalyst was highly stable for 20 h of reaction without a decrease in CH_4_ conversion for the CO_2_ reforming reaction.

**Figure 10:**
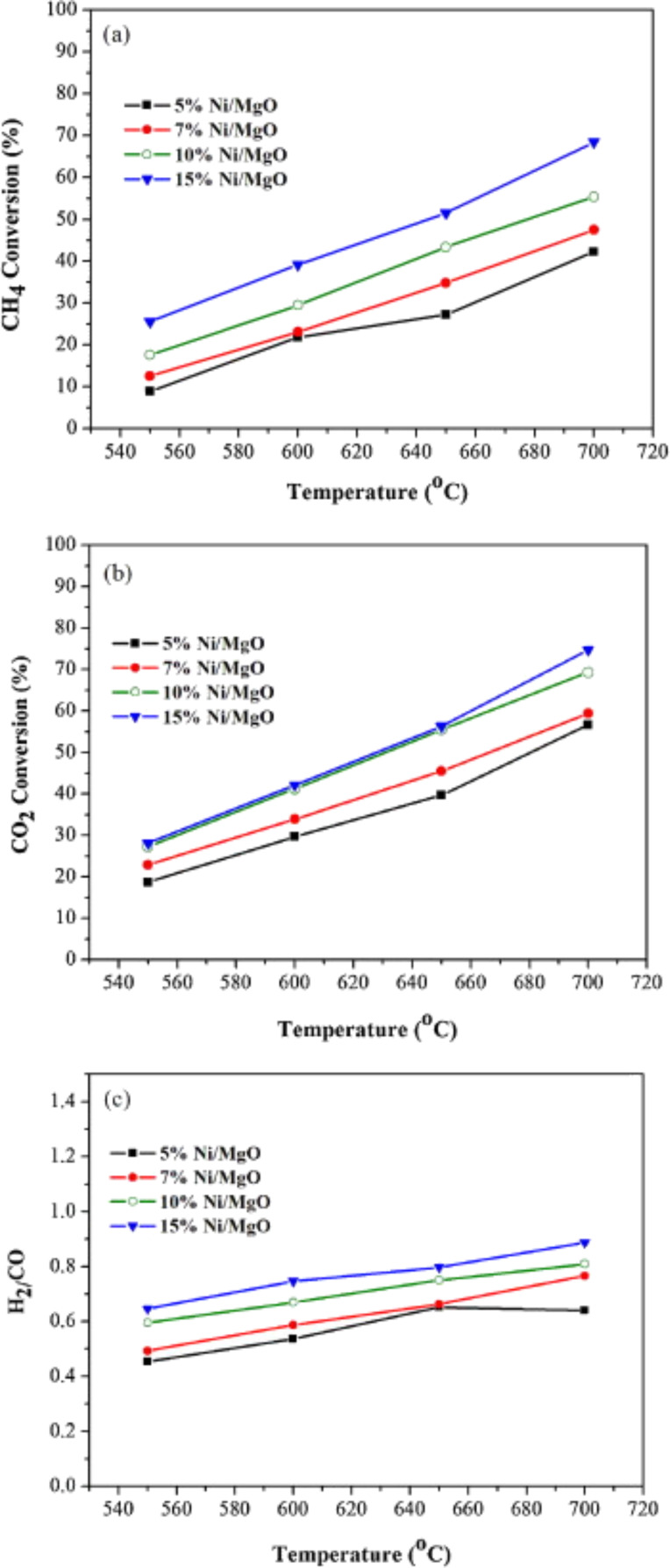
(a) CH_4_ conversion, (b) CO_2_ conversion, (c) H_2_/CO ratio of catalysts with different nickel loadings in the DRM reaction, CH_4_/CO_2_ 1:1 and gas hourly space velocity (GHSV) of 1.8 × 10^4^ mL/h·g_cat._ Reprinted with permission from [108], copyright 2014 Elsevier.

Meshkani et al. [111] studied the effect of Ni loading on the catalytic activity of a Ni/MgO catalyst in the DRM reaction. The CO_2_ and CH_4_ conversion, H_2_/CO ratio, and H_2_ and CO yields showed a progressive increase as the Ni loading increased from 5 wt % to 7 wt %. In contrast, the conversion yields decreased with higher Ni loading of 10 and 15 wt %. The conversion yields decreased because of low Ni dispersion. Meanwhile, the H_2_/CO ratio was reduced due to the occurrence of RWGS. In terms of stability, carbon deposition was observed at the Ni/MgO catalyst with 15 wt % Ni loading. However, this deposition could not deactivate the catalyst, and high stability was recorded up to a reaction time of 300 min. Furthermore, the reduced NiO–MgO solid solution catalysts could inhibit carbon deposition owing to the strong synergistic effect between high Ni dispersion and basicity of the MgO support. In contrast, Meshkani et al. [108] reported that low Ni loading results in low conversion of CO_2_ and CH_4_ over the Ni/MgO catalyst as shown in Figure 10. This finding is due to fewer NiO species being reduced to metallic species, thus minimizing the amount of active sites for the reaction. Stable conversion of CO_2_ and CH_4_ at 70% and 55%, respectively, were observed for the Ni/MgO catalyst with 5 wt % of Ni loading for long-term reactions up to 50 h.

**Figure 11:**
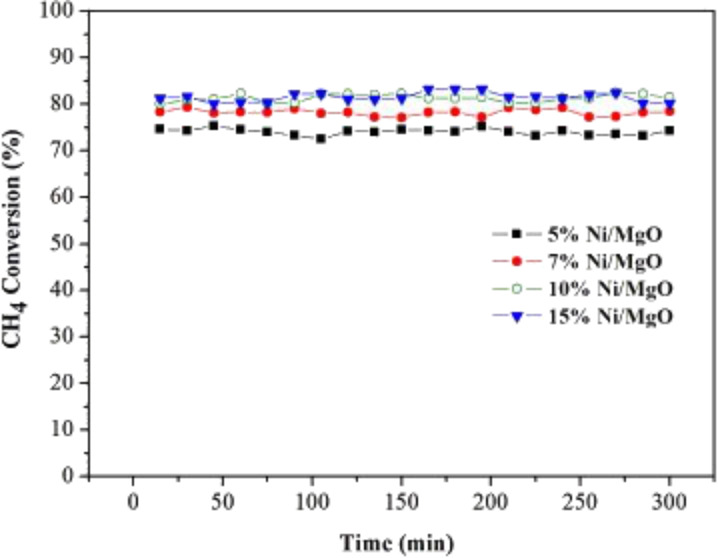
Stability of CH_4_ conversion over Ni/MgO catalysts with different Ni loadings (5% to 15%) in a DRM reaction at 700 °C, CH_4_/CO_2_ 1:1, and gas hourly space velocity (GHSV) of 1.8 × 104 mL/h·g_cat_. Reprinted with permission from [108], copyright 2014 Elsevier.

The high stability is due to the high basicity of the support and the formation of solid solution, thus inhibiting carbon deposition. Figure 11 shows the stability of the DRM reaction on the Ni/MgO catalysts with various nickel loadings at 700 °C.

Lin et al. [109] inspected the effect of Ni loading (2–20 wt %) on the Ni/MgO (111) catalyst prepared via the impregnation method. According to the morphology results, the 6 wt % Ni/MgO (111) catalyst showed a high Ni dispersion of 21.3% and a small particle size of 4.7 nm. The catalytic performance of the Ni/MgO(111) catalyst increased as the Ni loading increased from 2% to 10%; however, adverse effects were observed for the applied catalyst with 20% Ni loading. This finding may be explained by the decrease in metal dispersion and formation of large particles when the highest Ni loading was employed. Moreover, the 2 wt % Ni/MgO (111) catalyst produced a small particle size catalyst and was more easily oxidized during the reaction, possibly causing catalyst sintering. Meanwhile, Ni loading exceeding 6% may result in a larger particle size and poor oxidizability, thereby yielding high stability and consequent carbon deposition.

**Figure 12:**
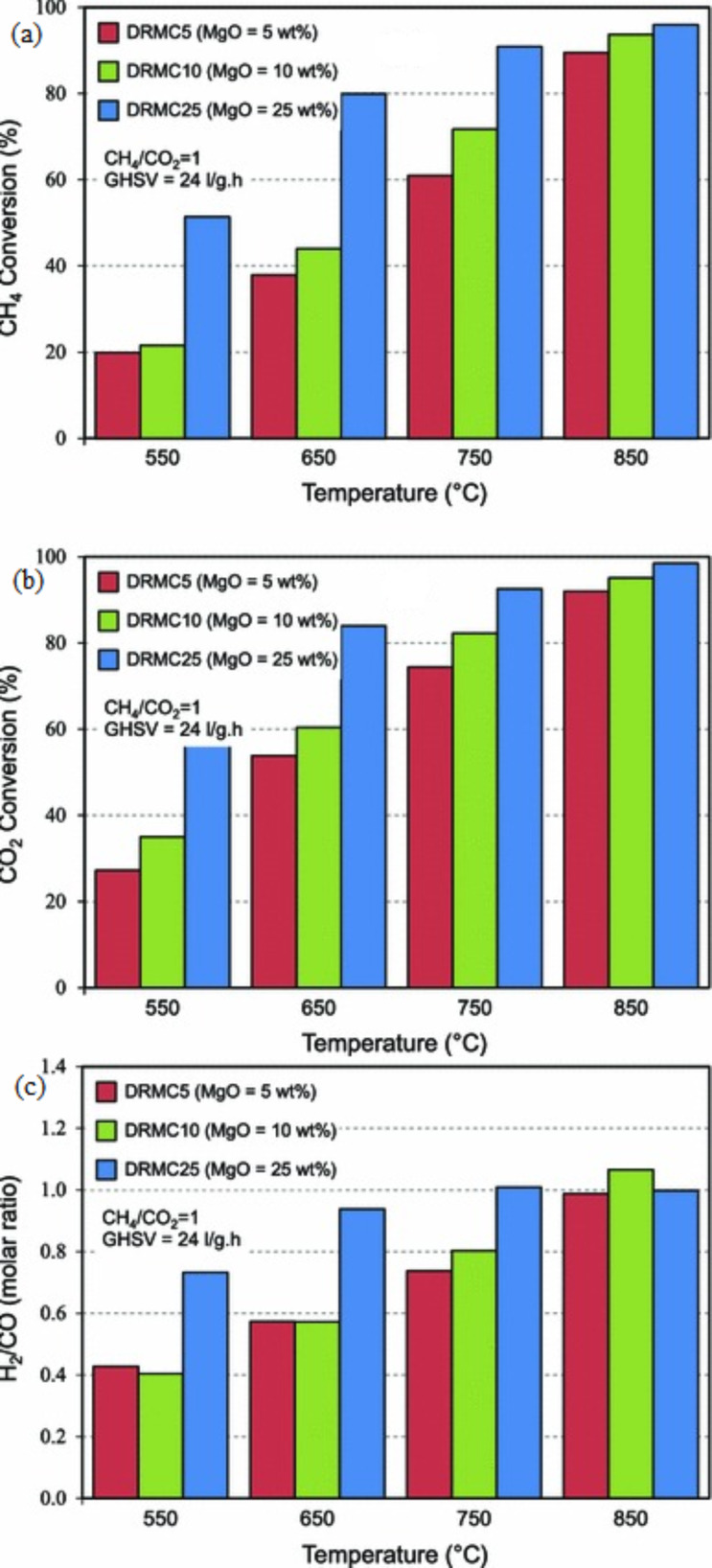
Effect of MgO loading on (a) CH_4_, (b) CO_2_ conversion and (c) H_2_/CO molar ratio over various MgO loadings (5, 10, 25 wt %). Reprinted from [38], copyright 2014, Springer Nature.

Sajjadi et al. [38] investigated the influence of MgO loading to feed conversion, product yield, H_2_/CO ratio, and stability over a Ni–Co/Al_2_O_3_–MgO–ZrO_2_ catalyst. The findings showed that the feed conversion increased as the metal loading of MgO increased. Notably, a 25 wt % of MgO loading showed the highest conversion of CO_2_ and CH_4_ with all applied reaction temperatures ranging from 550 °C to 850 °C as exhibited in Figure 12. This finding indicates a small particle size distribution, good morphology, and high surface area of the catalyst, as well as high diffusion of active metal without agglomeration during the reaction. Moreover, the average particle size was 11.6 nm, which was small enough to suppress carbon formation. Meanwhile, the product yield appeared to be markedly affected by the increment in MgO loading. The optimum yield of H_2_ and CO of 96.9 and 97.1, respectively, were recorded over the Ni–Co/Al_2_O_3_–MgO–ZrO_2_ catalyst containing 25 wt % MgO loading at a reaction temperature of 850 °C. This excellent performance is due to the properties of MgO, which provides a highly alkaline surface for CO_2_ adsorption. The stable yield with a H_2_/CO ratio close to unity was observed with the Ni–Co/Al_2_O_3_–MgO–ZrO_2_ catalyst with 25 wt % MgO loading. The catalyst was highly stable in the 24 h reaction time without deposition of carbon. MgO promotion enhanced the metal distribution when exposed to the highly basic surface and decreased the particle size.

Min et al. [104] studied the effect of Mg/Al ratio on catalytic activity and stability over a Ni–MgO–Al_2_O_3_ catalyst and found that the conversion of CO_2_ and CH_4_ showed improved results when MgO was introduced. The best catalytic performance was detected for a catalyst with a ratio of MgO/(MgO+Al_2_O_3_) of approximately 0.44 to 0.86. This outcome is due to the capability of MgO in enhancing the interaction between metal and support. Moreover, the compound improved the alkalinity of the catalyst surface, leading to increased adsorption of CO_2_. They proposed that the adsorbed CO_2_ reacted with deposited carbon to stimulate the catalyst. In a different study by Zhu et al. [112], different Mg/Al molar ratios of 5:1, 3:1, 1:1, 1:3, and 1:5 were applied for the preparation of a NiMg*_x_*Al*_y_* catalyst. The optimum Mg/Al molar ratio of 1:1 yielded the highest CO_2_ and CH_4_ conversions of 90% and 83%, respectively. These results are due to the formation of the hydrotalcite precursor for the NiMgAl catalyst. Moreover, MgO played an important role for improved catalyst performance; thus, a high Mg/Al molar ratio corresponded to the highest conversion in the order of NiMg_5_Al_1_ > NiMg_3_Al_1_ > NiMg_1_Al_3_ > NiMg_1_Al_5_. Furthermore, low carbon formation of 3.3 mg/g_cat_·h was observed with the NiMg_5_Al_1_ catalyst. Overall, the formation of hydrotalcite and a high ratio of Mg could improve the performance of Ni metal and enhance the catalytic activity and stability.

Alipour et al. [122] successfully investigated the effect of alkaline earth metals, such as MgO, CaO, and BaO, as support modifiers for a Ni/Al_2_O_3_ catalyst. The addition of basic support modifiers resulted in the reduction of the surface area of the Ni/Al_2_O_3_ catalyst. However, these modifiers could enhance the catalyst performance and inhibit carbon deposition. Interestingly, MgO showed an excellent effect on catalytic activity and carbon deposition. Furthermore, they analyzed the effect of different MgO loadings (1.5%–6%) on the Ni/Al_2_O_3_ catalyst. A moderate amount of MgO, that is, 3 wt %, was suitable for the Ni/Al_2_O_3_ catalyst because of stable catalytic activity and H_2_/CO ratio. Yu et al. [113] studied the effect of La as a promoter to a NiMgAl catalyst and found that the addition of La enhanced the basic surface of the catalyst and improved Ni metal distribution. Moreover, the catalytic activity increased, and the stability of the NiMgAl catalyst considerably improved. The combination of La and Mg suppressed carbon formation. The investigation using a La molar ratio ranging from 0.04 to 0.15 yielded the highest catalytic activity over the Ni_0.5_Mg_2.5_Al_0.9_La_0.1_O_4.5_ catalyst.

#### Effect of catalyst pretreatment

Pretreatment, such as calcination, is mainly conducted to eliminate and volatilize various catalyst precursors that are used during catalyst preparation, including hydroxides, nitrates, or carbonates, which are unnecessary for the final catalyst. High temperature treatment is typically conducted for this elimination. Notably, overheating of the solid catalyst results in high pressure because of trapped H_2_O molecules in the micropores, which may crack particulate carriers [95]. Feng et al. [94] investigated the influence of calcination temperature (between 600 °C to 800 °C) on the adsorption and dissociation of CO_2_ by applying DRM over the NiO/MgO catalyst. High performance and strong interactions between metal and support were observed with the calcination of the NiO/MgO catalyst at 800 °C. In addition, the catalyst calcined at 800 °C exhibited more active sites, which are strong enough to absorb CO_2_ on the metal surface. Moreover, CO_2_ was not directly adsorbed on the Ni metal, and dissociated H from CH_4_ cracking assisted the dissociation of CO_2_ into CO. This result was in an agreement with Wang et al. [23]. Wang et al. [23] stated that the activity and stability of the NiO/MgO catalyst was strongly dependent on calcination temperature. The calcination temperature influenced the metal/support interaction, which performs an important role in hindering sintering and carbon deposition. Moreover, the strong Lewis base property of MgO enabled the adsorption of more CO_2_ on the catalyst, consequently enhancing the conversion of reactants and minimizing the carbon deposition via a reverse Boudouard reaction (Equation 12).

**Figure 13:**
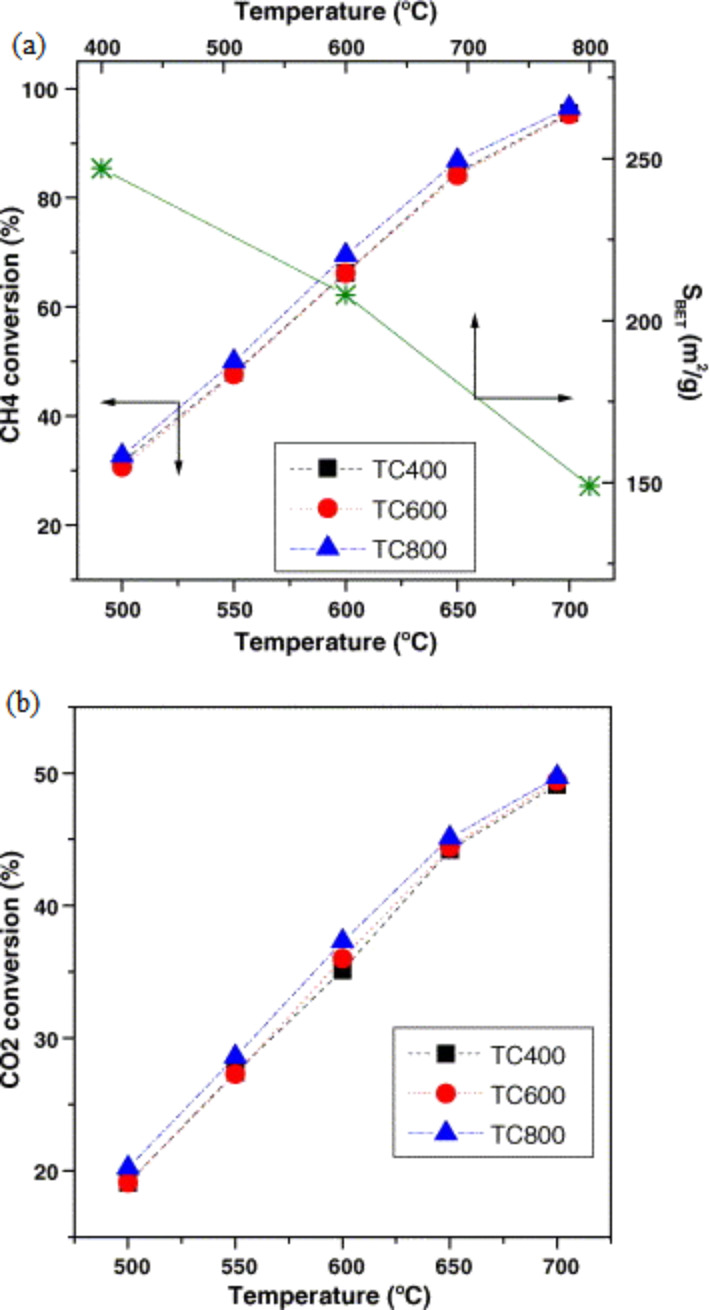
Conversion of methane (a) and carbon dioxide (b) on N55M11 calcined at different temperatures (■) 400 °C, (●) 600 °C and (▲) 800 °C and reduced at 700 °C. *S*_BET_ as a function of the calcination temperature. Reprinted with permission from [92], copyright 2006 Elsevier.

In contrast, Perez-Lopez et al. [92] studied the effect of calcination temperature (400 °C, 600 °C, and 800 °C) in a DRM reaction over a Ni–Mg–Al catalyst. They stated that the increase in calcination temperature from 400 °C to 800 °C yielded a decrease in surface area values from 250 m^2^/g to 150 m^2^/g, respectively. Figure 13a,b shows the conversion of CO_2_ and CH_4_ of the NiMg (loading of Ni 55 mol %; Mg 11 mol %) catalyst influenced by calcination temperature. As evidently seen from the results, the catalyst activity was independent of calcination temperature. However, significant differences in catalyst activity were observed, possibly as a result of the surface area of the catalyst. Moreover, this catalyst displayed high suppression of coke formation because of the synergetic effect of Ni and Mg, as proven by the highest concentration of basic sites obtained from the sample with lower Mg compared with Ni contents. However, they proposed a Ni/Mg ratio of lower than 5 for the best result. These results further highlighted higher carbon deposition for the catalyst calcined at 400 °C compared with those at other temperatures.

Serrano-Lotina et al. [107] investigated the influence of calcination temperature of a La–NiMgAl catalyst for biogas reforming. The catalyst was calcined at different temperatures between 250 °C and 750 °C. As a result, the grain size and cell parameter values increased with calcination temperature. The calcination temperature is extremely crucial, possibly affecting the activity and stability of the catalyst, as evidently proven by the significant decrease in activity at higher calcination temperature. An increased interaction inside the Mg(Ni,Al)O solid solution leads to agglomeration, which is inefficient for the catalytic reaction. The optimized calcination temperature was 550 °C for the La–NiMgAl catalyst to yield large catalytic activity. However, the La–NiMgAl catalyst calcined at 750 °C was reportedly the most preferable for coke suppression. To enhance catalytic performance, Son et al. [54] performed steam pretreatment over a NiCoMg/Al_2_O_3_ catalyst. During the treatment, MgAl_2_O_4_ was formed on the catalyst when the unstable aluminum leached out and moved to MgO. Aluminum leaching stimulated the acidic sites, leading to carbon deposition. Thus, the formation of MgAl_2_O_4_ on the catalyst enhanced the CO_2_ adsorption or desorption and consequently suppressed carbon deposition. From this study, long-term stability was achieved for 3000 h at a reaction temperature of 850 °C with high selectivity of H_2_ (93.8%) and CO (95.5%) and low carbon deposition (1.3 wt %) at a rate of 0.0003 (mg_c_/g_cat_·h).

#### Influence of reaction conditions

**Figure 14:**
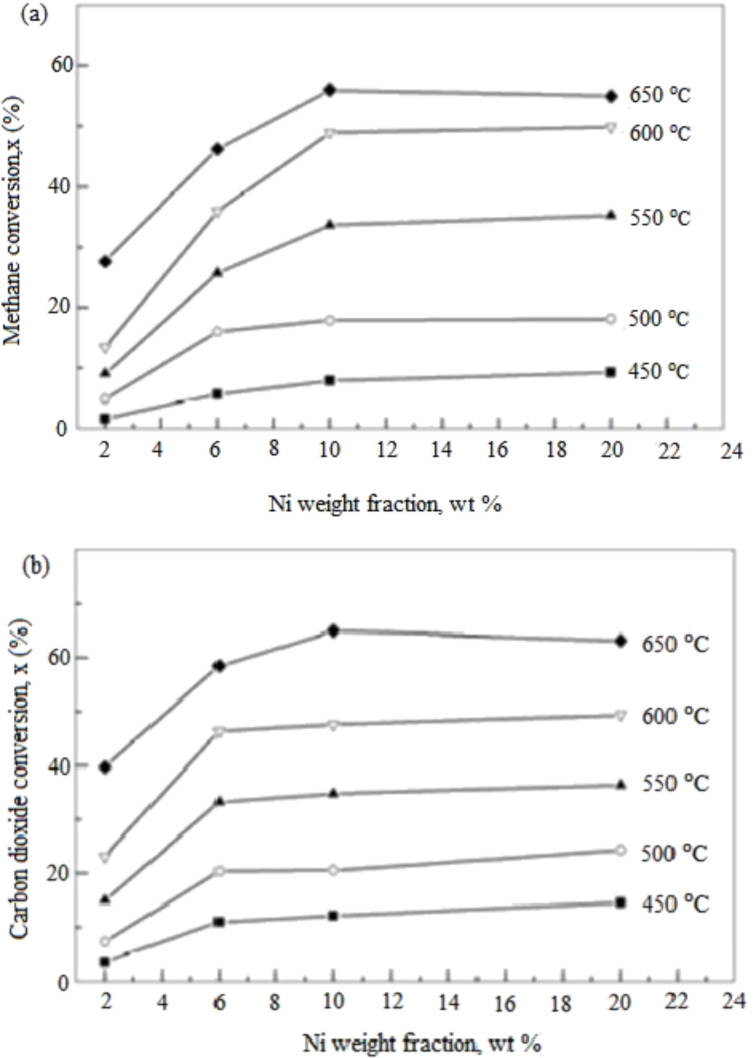
The effect of reaction temperature on catalyst performance: (a) CH_4_ conversion, (b) CO_2_ conversion, at feed ratio CH_4_/CO_2_ 1:1, gas hourly space velocity (GHSV) of 36000 mL/h·g, *P =* 1.01 × 10^5^ Pa. Reprinted with permission from [109], copyright 2015 Elsevier.

Meshkani et al. [108] carried out a DRM reaction at various temperatures from 500 °C to 700 °C for various Ni loadings on a Ni/MgO catalyst. The catalytic activity and H_2_/CO ratio were significantly affected by Ni loading ranging between 5–15 wt % as the reaction temperature increased. Excellent catalyst performance was recorded for the Ni/MgO catalyst with 15 wt % Ni loading. Lin et al. [109] investigated the influence of the reaction temperature of 450 °C to 650 °C for various Ni loadings as displayed in Figure 14. The study found that the CO_2_ and CH_4_ conversions gradually increased with a reaction temperature of up to 650 °C. The highest conversion of CO_2_ and CH_4_ of 65% and 60%, respectively, was recorded at a reaction temperature of 650 °C.

Meshkani et al. [111] investigated the effect of CO_2_/CH_4_ feed ratios of 3:1, 2:1, 1:1, and 1:2 on the catalytic activity and H_2_/CO ratio over a 5 wt % Ni/MgO catalyst at a reaction temperature of 700 °C. Table 5 shows the effect of feed ratio on the catalytic performance over a 5% Ni/MgO catalyst in the DRM. It could be concluded that the conversion of CH_4_ increased considerably as the feed ratio increased. However, the CO_2_ conversion and H_2_/CO ratio showed adverse effects.

**Table 5:** Influence of feed ratio on the catalytic performance of 5 wt % Ni/MgO catalysts in DRM reaction at 700 °C and gas hourly space velocity (GHSV) of 1.0 × 10^4^ mL/h·g_cat_ [111].

Feed ratio CO_2_/CH_4_	CH_4_ conversion (%)	CO_2_ conversion (%)	H_2_/CO

3:1	97.2	62.4	0.58
2:1	89.3	69.3	0.65
1:1	81.6	83.8	0.80
1:2	67.5	89.5	0.97

Meshkani et al. [108] further studied the effect of CO_2_/CH_4_ feed ratios of 1:2, 1:1, 1.5:1, and 2:1 on a 10 wt % Ni/MgO catalyst as shown in Table 6. The same pattern as in their previous study in [111] was observed. The highest CH_4_ conversion of 86.29% was obtained at a CO_2_/CH_4_ feed ratio of 2:1. Meanwhile, the highest CO_2_ conversion and H_2_/CO ratio of 81.95% and 0.91, respectively, were obtained at CO_2_/CH_4_ feed ratio at 1:2. Mirzaei et al. [123] found that CH_4_ conversion increased as the CO_2_/CH_4_ feed ratio increased from 0.5 to 2 over a 10 wt % CoMgO catalyst. However, the CO_2_ conversion and H_2_/CO ratio decreased with increasing CO_2_/CH_4_ feed ratio.

**Table 6:** Influence of feed ratio on the activity of a 10 wt % Ni/MgO catalyst in the DRM reaction at 700 °C and gas hourly space velocity (GHSV) of 1.8 × 10^4^ mL/h·g_cat_ [108].

Feed ratio CO_2_/CH_4_	CH_4_ conversion (%)	CO_2_ conversion (%)	H_2_/CO

1:2	60.42	81.95	0.91
1:1	69.07	77.35	0.80
1.5:1	78.40	66.13	0.77
2:1	86.29	59.56	0.64

**Figure 15:**
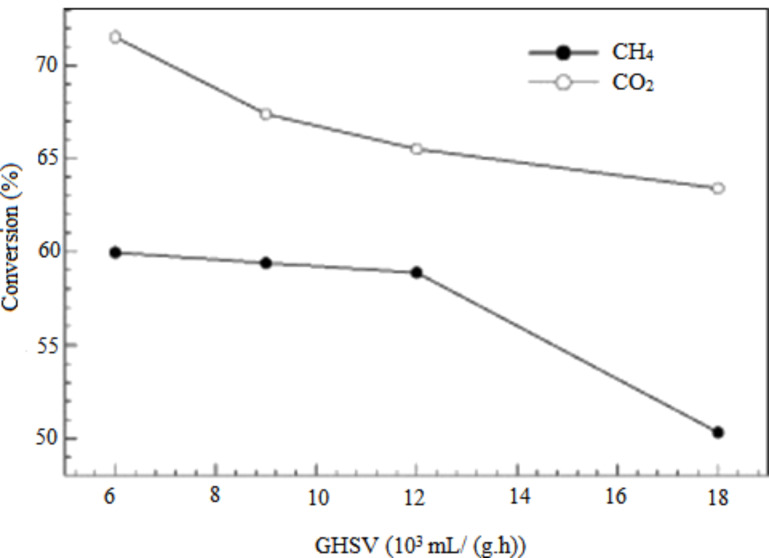
Influence of gas hourly space velocity (GHSV) on the catalyst performance of 20 wt % Ni/3 wt % MgO–Al_2_O_3_ catalyst at a feed ratio CH_4_/CO_2_ 1:1, *T* = 650 °C. Reprinted with permission from [70], copyright 2014 Elsevier.

The catalytic performance of a Ni/MgO–Al_2_O_3_ catalyst was examined for the application of various gas hourly space velocity measurements between 6 and 18 L/h·g_cat_ by keeping the reaction temperature and feed ratio in the system constant (*T* = 650 °C, CH_4_/CO_2_ 1:1) [70]. The CO_2_ and CH_4_ conversion decreased as the space velocity increased, as exhibited in Figure 15. This is due to the insufficient contact time for the reactant on the surface of catalyst, which leads to a decrease in catalytic activity.

Meshkani et al. [108] also reported the reduction of CO_2_ and CH_4_ conversion yields as the space velocity increased from 6 L/h·g_cat_ to 24 L/h·g_cat_ over a 10 wt % Ni/MgO catalyst. Table 7 displays the effect of gas hourly space velocity (GHSV) on the activity of a 10 wt % Ni/MgO catalyst in a DRM reaction at 700 °C and molar ratio of CH_4_/CO_2_ 1:1. The pattern of the CH_4_ conversion versus space velocity appeared similar to that of the study by Mirzaei et al. [123] over Co–MgO. Khajenoori et al. [110] investigated the space velocity of 6–18 L/h·g_cat_ on 10 wt % Ni–7 wt % CeO_2_/MgO catalyst at a reaction temperature of 700 °C and a feed ratio of CH_4_/CO_2_ 2:1. Conversions of CO_2_ and CH_4_ showed a negative effect with increasing gas hourly space velocity. This phenomenon was in agreement with the findings obtained by Meshkani et al. [108].

**Table 7:** Effect of gas hourly space velocity (GHSV) on the activity of a 10 wt % Ni/MgO catalyst in DRM reaction at 700 °C and molar ratio of CH_4_/CO_2_ 1:1 [108].

GHSV 10^4^ (mL/h·g_cat_)	CH_4_ conversion (%)	CO_2_ conversion (%)

0.6	83.75	86.77
0.9	80.16	84.33
1.2	75.60	80.36
1.5	66.14	70.75
1.8	63.36	68.99
2.4	58.88	64.61

## Conclusion

This review reported on the mechanism reaction for the DRM, mechanism of carbon formation and removal, development of Mg-containing oxide catalysts, and the effect of reaction conditions on catalytic performance. This work can be used as a reference for future works on the improvement of catalytic activity, selectivity, and stability. Through the mechanism, researchers can understand the reactions occurring in DRM, carbon deposition, and catalyst regeneration. This understanding can assist researchers in designing new multifunctional catalysts for DRM.

Two main issues requiring increased attention in catalyst development include pretreatment and the preparation method of the catalyst. First, in the catalyst preparation method for Mg as a promoter, the sol–gel method is more preferable, whereas cold plasma is highly suitable for synthesizing Mg as a support catalyst. Secondly, this review focused on the calcination temperature for catalyst pretreatment. Most researchers applied a high temperature range of 500 °C to 800 °C for markedly improving catalytic activity and minimizing carbon deposition. The analysis of the effect of reaction temperature on catalytic performance suggested that high temperatures of 800 °C and 950 °C were suitable for high CO_2_ and CH_4_ conversion reaching 99% and could inhibit carbon deposition. Given that methane decomposition and the Boudouard reaction contribute to carbon formation, a feed ratio (CH_4_/CO_2_) lower than unity should be used to suppress carbon deposition on the catalyst. All previous studies agree that the lowest space velocity is preferable for a DRM reaction to enhance catalytic activity. Optimization of metal loading for Mg-containing oxide catalysts also led to improved catalyst morphology and performance.

Despite considerable achievements by Mg-containing oxide catalysts as a support or promoter, the activity of Mg should be clearly specified and justified. Therefore, future investigations should focus on studying Mg-containing oxide catalysts at the molecular level to justify the capability of this highly basic catalyst for inhibiting carbon deposition in the dry reforming of methane reactions. Moreover, information on the effect of reduction on the prepared catalyst is lacking; thus, further research to analyze the effect of catalyst reduction on catalytic performance would prove highly beneficial. Reduction studies should include the composition of medium and time applied to the catalyst. Besides that, regeneration of the spent catalyst during dry reforming of methane reactions has yet to be studied extensively. This information would be extremely useful for improving catalyst performance and could be beneficial for decreasing the cost of preparing new dry reforming catalysts.
